# Detection of Error Related Neuronal Responses Recorded by Electrocorticography in Humans during Continuous Movements

**DOI:** 10.1371/journal.pone.0055235

**Published:** 2013-02-01

**Authors:** Tomislav Milekovic, Tonio Ball, Andreas Schulze-Bonhage, Ad Aertsen, Carsten Mehring

**Affiliations:** 1 Bernstein Center Freiburg, University of Freiburg, Freiburg, Germany; 2 Institute of Biology I, Faculty of Biology, University of Freiburg, Freiburg, Germany; 3 Department of Bioengineering, Imperial College London, South Kensington Campus, London, United Kingdom; 4 Department of Electrical and Electronic Engineering, Imperial College London, South Kensington Campus, London, United Kingdom; 5 Epilepsy Center, University Medical Center Freiburg, Freiburg, Germany; 6 Institute of Biology III, Faculty of Biology, University of Freiburg, Freiburg, Germany; Hospital Nacional de Parapléjicos, Spain

## Abstract

**Background:**

Brain-machine interfaces (BMIs) can translate the neuronal activity underlying a user’s movement intention into movements of an artificial effector. In spite of continuous improvements, errors in movement decoding are still a major problem of current BMI systems. If the difference between the decoded and intended movements becomes noticeable, it may lead to an execution error. Outcome errors, where subjects fail to reach a certain movement goal, are also present during online BMI operation. Detecting such errors can be beneficial for BMI operation: (i) errors can be corrected online after being detected and (ii) adaptive BMI decoding algorithm can be updated to make fewer errors in the future.

**Methodology/Principal Findings:**

Here, we show that error events can be detected from human electrocorticography (ECoG) during a continuous task with high precision, given a temporal tolerance of 300–400 milliseconds. We quantified the error detection accuracy and showed that, using only a small subset of 2×2 ECoG electrodes, 82% of detection information for outcome error and 74% of detection information for execution error available from all ECoG electrodes could be retained.

**Conclusions/Significance:**

The error detection method presented here could be used to correct errors made during BMI operation or to adapt a BMI algorithm to make fewer errors in the future. Furthermore, our results indicate that smaller ECoG implant could be used for error detection. Reducing the size of an ECoG electrode implant used for BMI decoding and error detection could significantly reduce the medical risk of implantation.

## Introduction

Even though the control of prosthetic devices using brain-machine interfaces (BMIs) has highly improved during the last several years [1]–[3], such devices are still prone to decoding errors. Decoding errors can elicit error related neuronal responses (ERNRs). Detecting these errors can be beneficial for the BMI performance. If detected, errors can be subsequently corrected, recognizing that a certain effector movement was not intended. This strategy has already been implemented in on-line BMI studies [4]–[7], but only in externally paced BMIs. However, many powerful BMIs are used to continuously decode and control the movements of an effector. Most prominent examples are the BMI control of a prosthetic arm [1], [8]–[10] and the brain control of a computer cursor [3], [9], [11].

Error detection can also be used to modify the decoding algorithm to make fewer decoding errors in the future. This approach is especially suitable for BMIs decoding continuous movements, since subjects correct for movement discrepancy by producing corrective movements, thereby making subsequent error correction obsolete. However, the feasibility of this strategy has so far been demonstrated only by computer simulations [12], [13].

**Figure 1:**
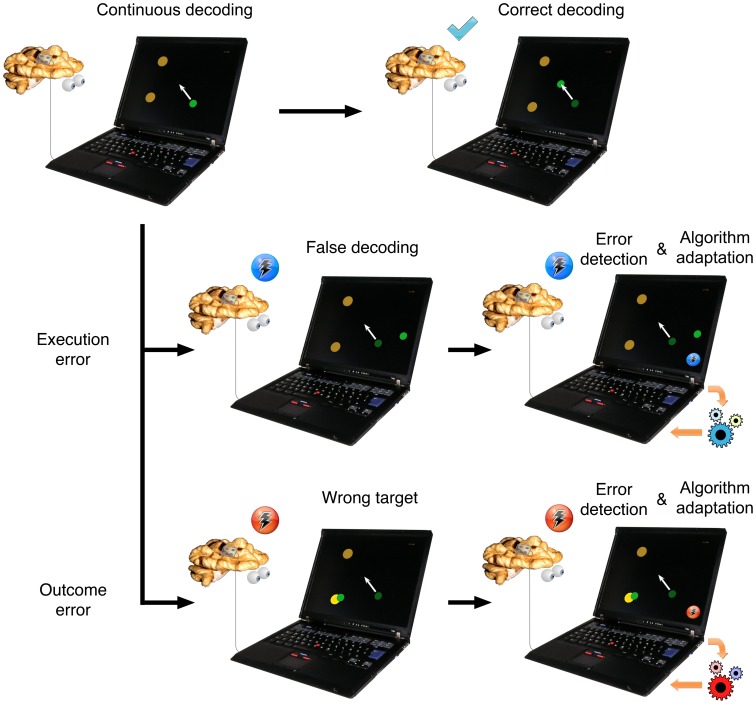
Application of neural activity based error detection for improvement of a continuous BMI control. Subjects intend to move a cursor towards the top left target (white arrow). If the decoding is correct, the cursor performs the intended movement and no neuronal error signal is elicited in a subject. If there is a discrepancy between the intended and the decoded movement, an ERNR can be elicited. If the discrepancy is large enough, it can elicit an execution ERNR. If the execution error is detected by the BMI system, the decoding algorithm can be adapted to reduce the number of errors in decoding in the future. If the unwanted movement causes the cursor to reach an unwanted target, an outcome ERNR may be evoked. If the outcome ERNR is detected by the BMI system, it can change the decoding algorithm as well, this time in a different way.

To apply error detection to continuous BMI control, it is necessary to show that ERNR are indeed elicited during such tasks. A number of studies investigated neuroal responses to errors during continuous movement tasks, identifying neuronal activity related to three different error types: (i) target error [14], [15], event where the task environment goes through unexpected changes such as target jumps, (ii) execution error [14], [16], event where the ongoing motor commands result in an unexpected movement, and (iii) outcome error [16]–[19], event where the desired goal of a movement is not achieved. Outcome and execution errors are of special interest for BMI application, since both error events can occur during online BMI control. For example, an execution error can occur when the decoding algorithm decodes incorrect movements, thereby moving the cursor or the prosthesis in an undesirable direction (Figure 1). If the difference of intended and executed movements is large enough, it can evoke an execution ERNR. If the incorrectly decoded movement causes the effector to reach an unintended goal or perform an unintended function, this can elicit an outcome ERNR (Figure 1).

In recent years, electrocorticography (ECoG) emerged as a possible alternative to intracortical recordings as a recording technique that can be used for a continuous BMI [20]–[22]. Continuous BMI controlling two degrees of freedom has already been realized using ECoG [23]. Other recent studies showed that many other movement primitives can be decoded offline from ECoG signals, such as 7 degrees of freedom of arm movements [24], individual finger movements [25] and natural grasps [26]. Therefore, ECoG is a suitable platform for implementing a continuous BMI.

To use error signals in a ECoG based BMI, one needs to show that ERNRs can be detected from the ECoG signal with sufficient reliability. A recent study by Milekovic et al. [16] showed that both outcome and execution ERNR are present in human ECoG recordings during a continuous task. Here, we showed that the times of the events that elicited these ERNR can be detected with high accuracy.

## Methods

Experimental task, recording techniques and properties of the recoded data are described in detail in Milekovic et al. [16]. Here we provide only a short description.

### Task

**Figure 2:**
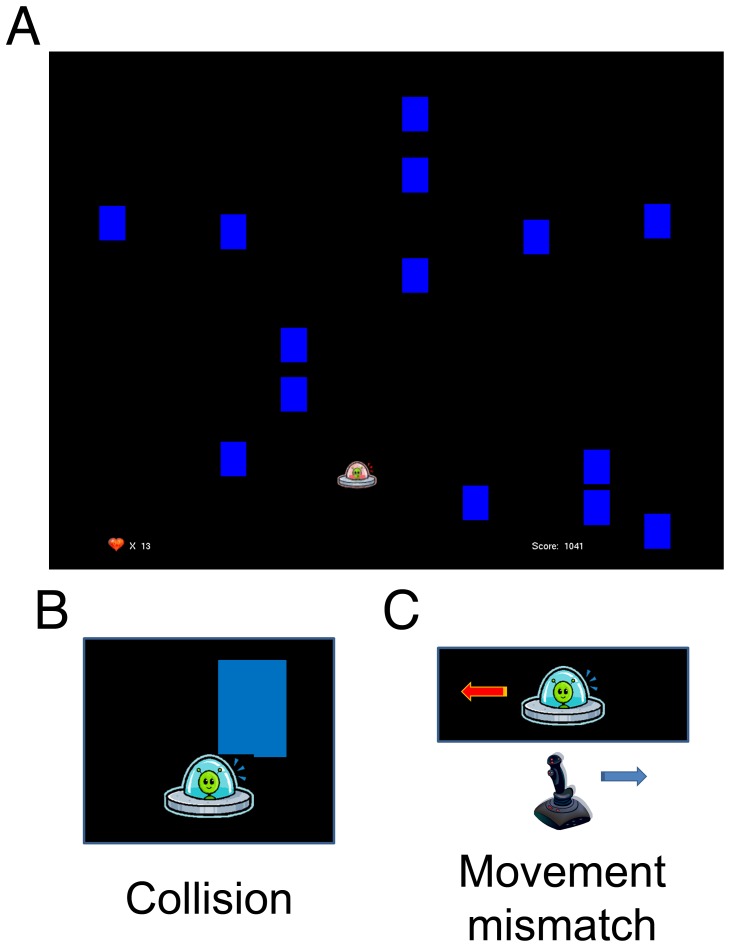
Task and error events. A: Screenshot of the paradigm as seen by the subjects on the computer screen. Subjects played a video game in which they moved a spaceship in the horizontal direction (left-right) to evade the blocks dropping from above. Every time the spaceship collided with a block (collision event; B) one life was lost. Collision events elicited outcome ERNR. From time to time, the spaceship moved in the opposite direction of the joystick movement for 500 ms (movement mismatch event; C). These events elicited execution errors.

Subjects (S1–S4) played a simple video game in which they controlled a spaceship with a small analogue joystick on a gamepad (Logitech® Rumblepad™ 2, Logitech Europe S.A., Morges, Switzerland) in the horizontal dimension (left-right; Figure 2a; Supplementary movie 1). The task was to evade blocks dropping from the top of the screen at a constant speed. The game was challenging enough so that the spaceship occasionally collided with a block (collision event, Figure 2b). At random moments, the spaceship moved in the direction opposite to the joystick movement for the duration of 500 ms (movement mismatch event, Figure 2c). Points were awarded for moving the spaceship, and subjects were instructed to gather as many points as possible. Subjects started the game with 20 “lives”. Each time the spaceship collided with a block, the number of “lives” was reduced by one. When the number of “lives” reached 0, the game, together with the recording session, ended. Recording sessions lasted between 5 and 24 minutes. We identified the neuronal responses to collision and mismatch events that were not mixed with neuronal responses to other events by defining a subgroup of “clean” outcome and “clean” mismatch events, consisting of events at least 2 seconds away from any other event of any kind. The total number of events recorded for each of the subjects is given in Table 1.

**Table 1:** Number of recorded sessions and events for each of the subjects.

Subjects	Sessions	Collision events		Mismatch events	
		All	Clean	All	Clean
S1	8	160	120	195	155
S2	4	80	38	227	185
S3	4	80	51	121	92
S4	6	120	87	71	38

To earn more points, subjects had to try to stay in the game as long as possible. Therefore, collision events presented a clear disadvantage in reaching the goal of the game. Thus, collision events reflected outcome errors. During the movement mismatch event, there was a clear discrepancy between the intended movement and the movement performed by the spaceship. Thus, movement mismatch events reflected execution errors.

In our previous study [16] we demonstrated that the ECoG signals we used for detection in the present study reflect ERNRs and are not movement related, nor related to visual stimuli or to surprise effects.

### Subjects and Recordings

Four subjects (3 male, 1 female) suffering from intractable pharmaco-resistant epilepsy voluntarily participated in the study after having given their informed consent. The study was approved by the Ethics Committee of the University Medical Center, Freiburg, Germany.

**Figure 3:**
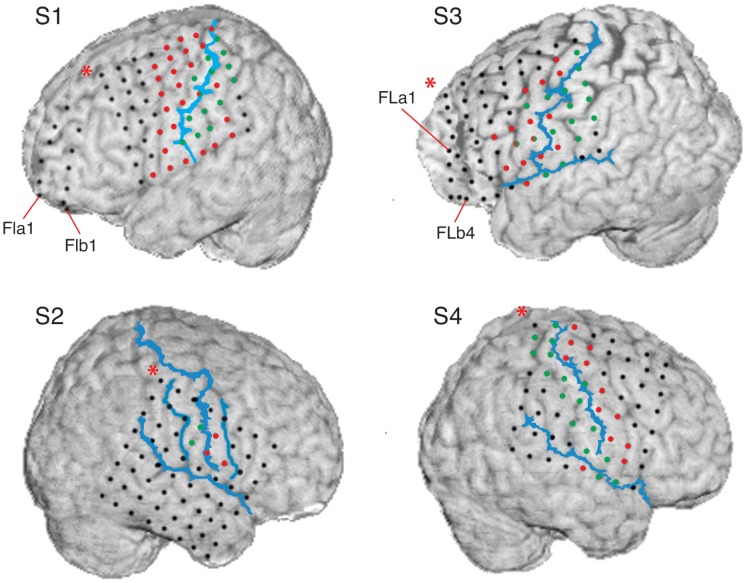
Locations of ECoG grid electrodes in relation to the cortex. Electrode positions (red, green and black circles) were reconstructed from the post-implantation MRI scan and positioned over the pre-implantation MRI scan [88]. For S1, S3 and S4 red (green) circles represent electrodes that showed motor (somatosensory) responses to electrical stimulation mapping (ESM). For S2, motor and somatosensory electrodes were determined from sulci reconstruction. Central sulci, Sylvian fissures and, for S2 only, pre and post central sulcus are shown as blue lines. These were drawn by hand to resemble sulci reconstruction from the post-implantation MRI scan. Each of the subjects was implanted with an 8×8 ECoG grid. In S2, no recordings were made from the top row of the ECoG grid. In addition to ECoG grids, the Figure shows two 6 electrode ECoG strips over the frontal lobe (FLa and FLb) for S1 and two 4 electrode ECoG strips (FBa and FBb) over the frontal lobe for S3. In this study, we analyzed the recording from the ECoG grids only.

For pre-neurosurgical epilepsy diagnosis, the subjects were implanted with an 8×8 grid of subdural surface electrodes covering parts of the primary and pre-motor cortex (Figure 3). Additional subdural surface and deep brain electrodes were implanted for subjects S1, S2 and S3 [16]. For all subjects, signals from 22 electrodes of EEG, two to four electrooculogram (EOG) electrodes, electrocardiogram (ECG) and electromyogram (EMG) were recorded simultaneously with the recordings made from the subdural and deep brain electrodes. Here, we analyzed the recordings from the 8×8 grid of subdural electrodes only.

Recordings from all electrodes were digitized at 256 Hz sampling rate for S1 and S2 and at 1024 Hz sampling rate for S3 and S4. No analogue filters were used during the data acquisition. Power line frequency was 50 Hz. Data analysis presented here was performed after the experiment using the MATLAB software package (MATLAB version 7.4–7.11, Natick, Massachusetts: The MathWorks Inc., 2007–2011).

### Measures of Detection Accuracy

Consider a process where a subject is actively observing a scene and, when a given stimulus appears, a neuronal response is elicited. Assume that neuronal activity is continuously recorded and a detection algorithm is continuously evaluating whether a stimulus appeared, given the neuronal activity. The efficiency of the detector can then be measured by comparing two point processes: the set of time points when the stimulus was presented and the set of time points when the detector detected the stimulus from the neuronal recordings.

Due to the internal processes in the brain and other sources of noise, even a perfect decoder will have a temporal noise in the detected times of events. On the other hand, detected events will still be useful, even if the times of detections are not perfectly aligned to the times of the events. For some applications, high temporal precision is not necessary. We describe this requirement on our detector as temporal tolerance.

If we tolerate the detected events within a time *Δt* from the real events, then any detection within this time window will be counted as a true positive detection. Every event window in which there are no detected events will be counted as false negative detection. For measuring the detection accuracy, we would also need to know the ability of the detector at predicting non-events. To obtain a fair estimate of such ability, the area between the event time windows has to be divided in windows of the same size, 2*Δt*. Every non-event time window in which there are no detected events will be counted as a true negative detection and every non-event time window in which there is a detected event will be counted as false positive detection.

#### Sensitivity and specificity of a detector

Accuracy of a detector can be described by measuring how well it performs in two different tasks: (i) detecting events when events are present and (ii) not detecting events when events are not present. One way to describe the first property is by measuring the sensitivity of the detector by calculating the true positive rate (*TPR*) [27] as a number of true positive detections (*N_TP_*) divided by the total number of real events. Since the total number of real events is given by the sum of true positive detections and false negative detections (*N_FN_*), the true positive rate is given by:
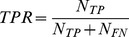
(1.1)


The second property can be described by measuring the specificity of the decoder by calculating the false positive rate (*FPR*) [27] as the ratio of false positive detections (*N_FP_*) divided by the number of all detections. Since the total number of all detections is given by the sum of true positive detections and false positive detections (*N_FP_*), the false positive rate is given by:
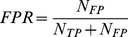
(1.2)


*FPR* definition used here should not be confused with the alternative definition of the false positive rate (*FPR_ALT_*) [28]:
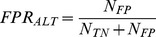
(1.3)


A disadvantage of measuring the detection accuracy by *TPR* and *FPR* is that one cannot directly compare two different detectors when both *TPR* and *FPR* of one detector are higher than the *TPR* and *FPR* of the other detector. Therefore, a metric incorporating both sensitivity and specificity of the detector is needed. One such metric is the mutual information.

#### Mutual information of a detector

One way to measure the performance of a detector is to calculate the mutual information [29] between a dataset containing times of real events and a dataset containing times of detected events. The mutual information is given by:
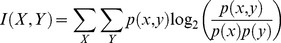
(1.4)where *X* and *Y* are the sets of all possible states of the real and detected event datasets and *x* and *y* are specific states from those sets, *p*(*x*) and *p(y)* are the probabilities of specific states and *p(x,y)* is the joint probability that states *x* and *y* occur jointly. In our case, the set of real event states consists of “real event” (*re*) and “real non-event” (*rne*), while the set of detected events consists of “detected event” (*de*) and “detected non-event” (*dne*). Joint and marginal probabilities used to calculate the mutual information are given by:



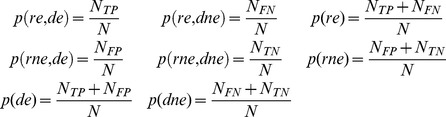
(1.5)Given a certain dataset of real event times and certain tolerance, the maximum value of the mutual information is obtained when detected event times perfectly match real event times. This value is identical to the entropy of real event times *H(X)*:
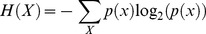
(1.6)


To compare the mutual information over different tolerances, we calculated the normalized mutual information, *C_YX_* [30]:
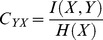
(1.7)


Calculating mutual information and entropy from a recorded dataset will give a good estimate of their true values, as long as the calculated probabilities are good estimates of the real probabilities. However, recorded datasets have a finite length, which will make the estimated probabilities fluctuate around their real values. Using the estimated probabilities to calculate the mutual information and entropy leads to a bias in the estimation [31]. To remove the bias of the mutual information, we used first and second order terms of the mutual information bias expansion derived in the study by Treves and Panzeri [31]:
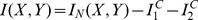
(1.8)

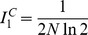
(1.9)

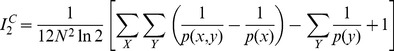
(1.10)where *I_N_(X,Y)* is the mutual information estimated from a dataset of length *N*. Here, the values of joint and marginal distributions have also been estimated from the same dataset. To remove the bias of the entropy, we used first and second order terms of the entropy bias expansion derived in the study by Victor [32]:
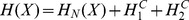
(1.11)

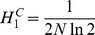
(1.12)

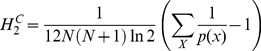
(1.13)


Thus, the bias corrected value of the normalized mutual information was calculated as:
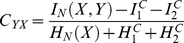
(1.14)


### Experimental Data Analysis

#### Preprocessing

Common-average referencing for grid electrodes was performed using all grid electrodes that showed no artefacts (one electrode for both S3 and S4 had to be excluded). To correct for changes in electrode recording offsets between sessions, the mean voltage over the entire session was subtracted for every session and for every electrode after re-referencing.

#### Signal components

**Figure 4:**
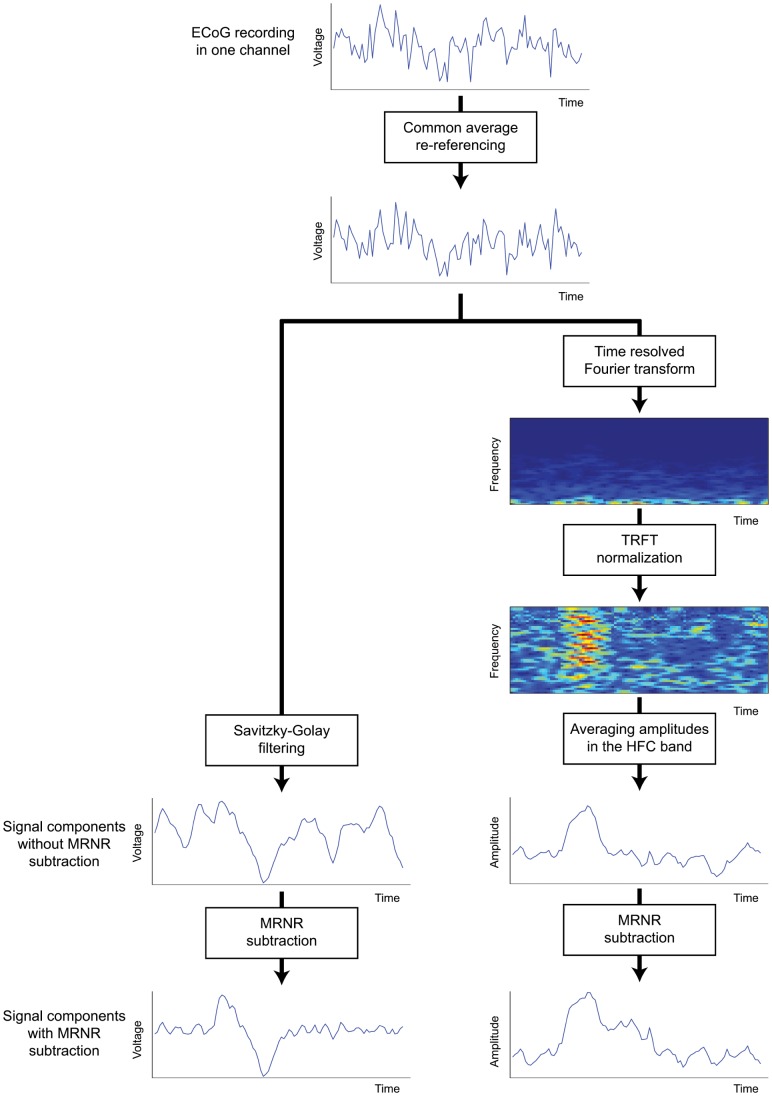
Extraction of low and high frequency components (LFC and HFC) from the ECoG recordings. Recordings in every channel were first re-referenced using the common average over all recording channels that do not show epileptic activity. To get the LFC, the re-referenced signal was low-pass filtered using a Savitzky-Golay filter (symmetric, 2^nd^ order, 250 ms window length). To get the HFC, the re-referenced signal is transformed to the time-frequency space using short-time Fourier transform. The amplitudes of the transformed signal were then divided by the average baseline amplitude for every frequency bin separately. The HFC was defined as the average normalized amplitudes across all bins within the HFC frequency range (see Methods for a definition of the frequency ranges).To correct for movement related neuronal activity, MRNRs were subtracted.

We analyzed the low and high frequency components of the recorded ECoG signals (Figure 4). The low frequency component was extracted by smoothing the preprocessed ECoG signals using a symmetric 2^nd^ order Savitzky-Golay filter [33], [34] with a time window of 250 ms (nominal 3 dB cut off frequency: 7.85 Hz for S1 and S2, 7.59 Hz for S3 and S4; estimated using table from [35]; for justification on using this filter see [16], section 1 of the supplementary material). We defined a window around each event of any kind, starting 3 seconds before the event and lasting until 3 seconds after it. The signals outside all of these windows were used as baseline activity. To enable a clear comparison to baseline, the average baseline activity was subtracted from the filtered recordings in each session for each electrode. The resulting signal was defined as the low frequency component of the signal (LFC).

To analyze the high frequency component of the signal, time-resolved Fourier transformation using a Hamming window (333 ms window width, shifted in steps of 31 ms) was applied to the preprocessed signals, and the amplitudes were used for further analysis. To account for the general decrease in amplitude with increasing frequency, the amplitudes of every frequency bin were normalized by dividing them by the average baseline amplitude of the same frequency bin in the respective session. We then extracted the average amplitude across a frequency band from 60 Hz to 128 Hz for S1 and S2 and from 60 Hz to 200 Hz for S3 and S4. Since recordings for subjects S1 and S2 were sampled at 256 Hz, spectral amplitudes could be calculated only for frequencies up to 128 Hz (Nyquist frequency). Therefore, the frequency band used to calculate high frequency component could only comprise frequencies up to 128 Hz for subjects S1 and S2. Amplitudes calculated from ECoG signals recorded at least 3 seconds away from any event were used as baseline. To enable a clear comparison to baseline, the average baseline activity was subtracted from the extracted amplitudes in each session for each electrode. The resulting signal was defined as the high frequency component of the signal (HFC).

When LFC and HFC were used together for detection, we normalized every electrode and signal component to zero mean and unit variance, to accommodate for their different scaling.

#### Detection algorithm

**Figure 5:**
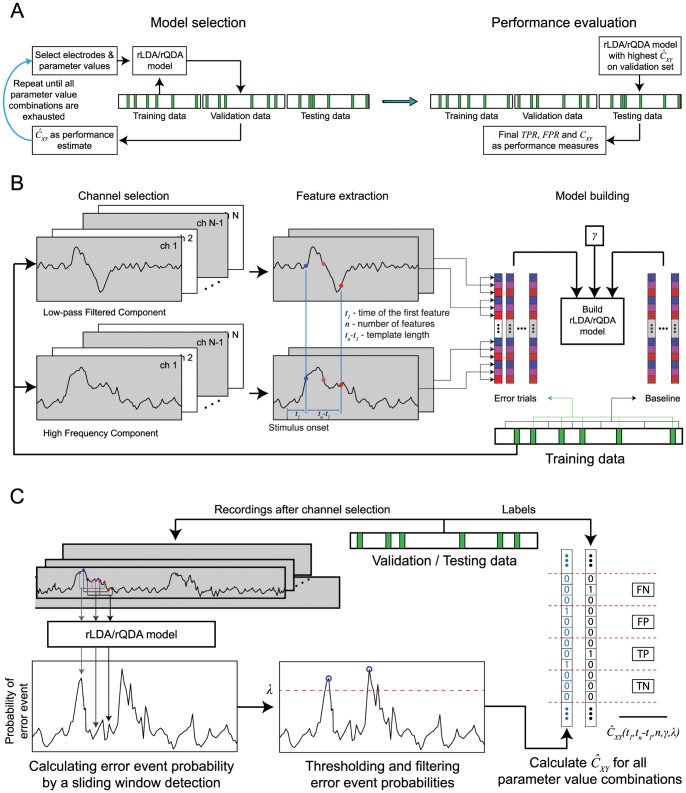
Classifier selection and performance evaluation of the error detection algorithm. (A) The dataset was divided into three parts of equal length: training, validation and testing. The training data set was used to build the detection algorithm. To optimize the parameters of the detection algorithm (see Methods), the detection performance was evaluated on the validation data set for different values of the parameters. The parameter values yielding the highest performance were used to build the detection classifier which was then applied on the testing data to evaluate its performance. (B) Classifier building: The classifier was built using signal features from error (green) and baseline epochs (white). Signal features were taken from LFC and HFC (Figure 4) at multiple time points and from multiple electrodes. (C) Performance evaluation: The classifier used to calculate probability of an error event in a sliding window fashion, across the continuous signals, in order to detect error events. Finally, performance was calculated by comparing the times of detected error events to the times of the real error events.

To detect error events from the neural activity, we trained a set of classifiers that captured the neuronal features which are specific to error events (Figure 5). To be sure that the training data did not include neuronal responses to non-error events which were erroneously identified as ERNRs, we used ERNRs elicited by “clean” events only. Given a signal component Φ, the peri-error feature vector was defined as:
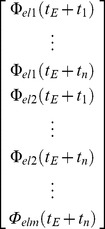
(1.15)where *t_E_* is the time of an error event, *el*1, *…, elm* are the selected electrodes and *t_1_, …, t_n_* are the selected time points relative to the time of the error event. Therefore, the feature vector contains *n•m* features for one signal component. If more than one signal component was used, the feature vectors of the signal component were concatenated, yielding an *l•n•m* dimensional feature vector, where *l* is the number of signal components used (*l* = 1 or 2 in this study). The time points *t_1_, …, t_n_* were always equidistant and defined by a set of parameters: (i) the time of the first feature in relation to the time of the error event, *t_1_*, (ii) the number of time points, *n* and (iii) the temporal distance between the first and the last feature, *t_n_-t_1_*.

Each classifier was build using two classes of feature vectors, the error class containing peri-error feature vectors (*E_class_*) and the baseline class containing feature vectors when no errors were present (*B_class_*).
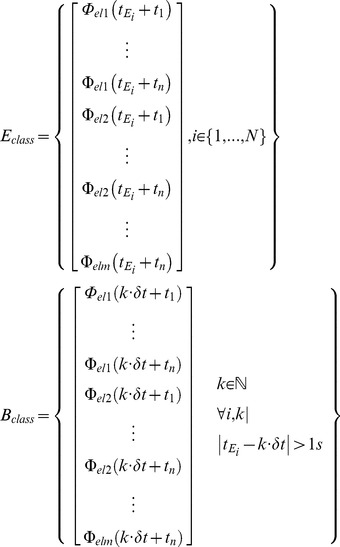
(1.16)where *δt* is the time resolution of the signal component (LFC: 4 ms for S1 and S2, 1 ms for S3 and S4; 31 ms when HFC or both components were used). ”

These classes were used to build either a regularized linear discriminant analysis (rLDA) classifier or a regularized quadratic discriminant analysis (rQDA) classifier [36]. A QDA classifier is built by fitting a Gaussian distribution to each of the classes and gives the probability to belong to one of the classes for any arbitrary point in the feature space. Each Gaussian is represented by a class mean and a covariance matrix. LDA is a simplification of QDA, where fitted Gaussian distributions share a common covariance matrix. Regularization is implemented by modifying the covariance matrix of the fitted Gaussian distributions and has the purpose to improve the accuracy of the discriminant analyses on a new, independent set of data. We used the regularization of the form:
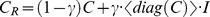
(1.17)where *C* is the covariance matrix of the fitted Gaussian distribution, *C_R_* is the regularized covariance matrix, *γ* is the regularization coefficient and *I* is the identity matrix of the same size as *C*. In the case of rQDA, the number of vectors in the baseline class is much higher than the number of vectors in the error class. Therefore, we regularized the covariance matrix of the Gaussian fitted to the error class only. The classifier was then used to calculate the probability of every feature vector *F* in the test dataset to belong to the error class.
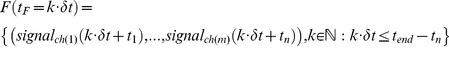
(1.18)where tF is the time point corresponding to the calculated probability and tend is the duration of the tested dataset. We then extracted all local maxima of the probabilities and assigned a ‘detected as non-error’ (dne) state to remaining points. A threshold value λ was then selected and we assigned a dne state to all time points for which the value of the maxima remained below the threshold. Finally, we assigned a dne state to all remaining maxima for which there was a higher maximum less than 1 second away. We assigned a ‘detected as error’ (de) state to all remaining maxima. Since the classifier should be able to detect error events for which ERNRs were only slightly mixed with neuronal responses to other events, we calculated the detection measures between the times of detected events and the times of all real events within the test dataset, instead of just using the “clean” events.

To properly validate the classifiers, we divided the recorded data into three similarly long parts by splitting each session into three parts, each containing one third of the “clean” events. First, we chose a set of parameter values consisting of: (i) a time of the first feature in relation to the error event, *t_1_*, (ii) a number of features, *n* and (iii) a time distance between the first and the last feature, *t_n_-t_1_*, (iv) regularization coefficient, *γ*, and (v) probability threshold *λ*. An rLDA or rQDA classifier was then built using the first part of the dataset. Using the built classifier, we detected the events on the second part of the data and calculated the *C_YX_*. Values of the parameters were then changed and the process was repeated, until all parameter values from the parameter grid were tested. We used the following grid of parameter values: (i) *t_1_*: from −667 ms to 667 ms in steps of 56 ms; (ii) *n*: 1, 3, 4, 5 and 8 when using single electrodes and electrode quartets for detection and 1, 2 and 3 when using anatomical electrode subsets or all grid electrodes for detection; (iii) *t_n_-t_1_*∶100 ms, 125 ms, 250 ms, 500 ms, 750 ms and 1000 ms; (iv) *γ*: 0, 0.01, 0.1, 0.3, 0.5, 0.7, 0.9, 0.99 and 1; and (v) probability threshold: from 0.5 to 1 in steps of 0.017. The classifier that gave the maximum *C_YX_* on the second part of the dataset was then used to detect the events on the third part of the dataset and *TPR*, *FPR* and *C_YX_* were then calculated from this detection result. The same process was repeated, now using the third part of the dataset for testing the grid of parameter values and the second part of the dataset for classifier testing. The average values of the two sets of *TPR*, *FPR* and *C_YX_* measures were then reported as the measured detection accuracy.

Different tolerance values were used to bin the experiment time into non-overlapping time bins, as described in section “Measures of detection accuracy”. The tolerance value directly determines the length of the dataset. Table 2 gives the dataset lengths for the tolerance values used.

**Table 2:** Total dataset length for different temporal tolerances.

Tolerance		50 ms	155 ms	261 ms	366 ms	472 ms	577 ms	683 ms	788 ms	894 ms	1 s
Dataset length	LFC	34 253	10 898	6 233	4 347	3 326	2 724	2 274	1 947	1 701	1 503
	HFC	31 041	9 892	5 665	3 953	3 030	2 482	2 076	1 780	1 557	1 381
	LCF & HFC	31 041	9 892	5 665	3 953	3 030	2 482	2 076	1 780	1 557	1 381

#### MRNR subtraction

To remove the movement related neuronal responses (MRNRs) following a mismatch or a collision event, we used the MRNR subtraction method successfully applied in our previous study [16]. MRNR were identified by deriving and testing a set of classifiers relating the signals from one electrode to the movements using only non-event data (i.e. all data which was at least 1 s before and 3 s after any event). The most predictive classifier was selected and used to predict the MRNR for the whole recording, this time including the event data. MRNR signals were then subtracted from the initial signal, and the result was termed MRNR-free signals. All reported results were achieved by using MRNR-free signal for detection, unless specified otherwise.

### Neuroanatomical Analysis

To determine whether the motor or the somatosensory cortex played a more distinctive role in generating ERNR, we classified electrodes as motor cortex electrodes, somatosensory cortex electrodes, and other electrodes (Figure 3) in the same way as done in the previous study by Milekovic et al. [16].

### Statistical Analysis

All results are reported as mean ±95% confidence interval. To calculate confidence intervals, we used a bootstrap method with 10 000 re-samples [37]. Every reported statistics was bootstrapped separately for each of the subjects. For instance, if the difference between *C_YX_* was reported, we bootstrapped the difference directly. Subject-wise values of the statistics were considered as independent measurements and reported values were calculated using the error weighted mean [38].
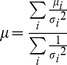
(1.19)

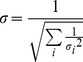
(1.20)where *µ_i_* and *σ_i_* are the subject-wise value of the statistics and its corresponding standard deviation estimated by bootstrapping, and *µ* and *σ* are the reported values of the statistics over subjects. We assumed that the measurements of the statistic were normally distributed and used the t-distribution to calculate the 95% confidence intervals [38].

## Results

### Detection of Error Related Neuronal Responses

**Figure 6:**
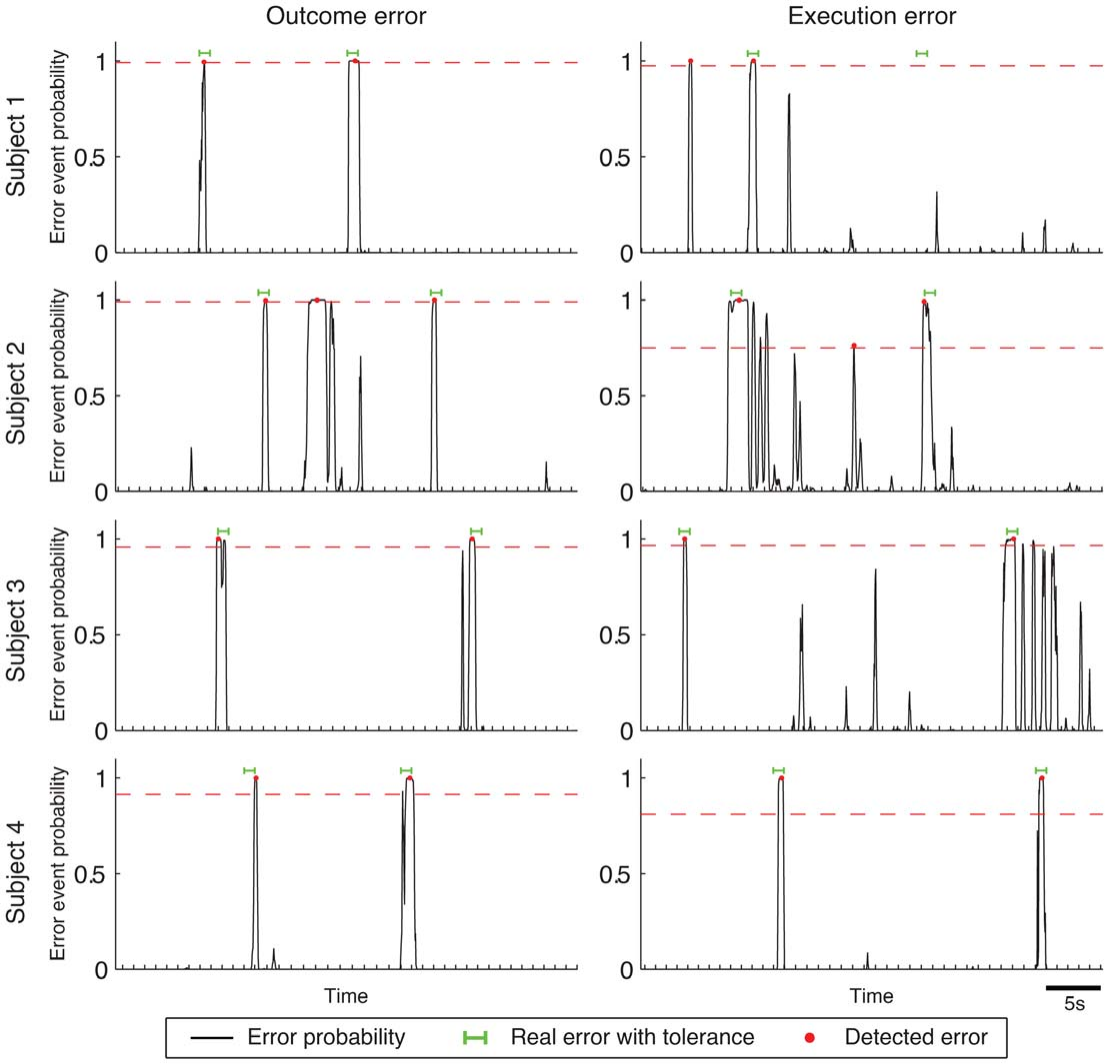
Examples of detector output time courses for each of the subjects. Probabilities of a detected error are shown together with the times of the detected events (red dots). The interval considered as correctly decoded error event using the tolerance of 366 ms is shown by green lines and the detection threshold by a dashed red line. Ticks on the time axis mark the borders of each of the time bins used to calculate the detection performance measures.

**Figure 7:**
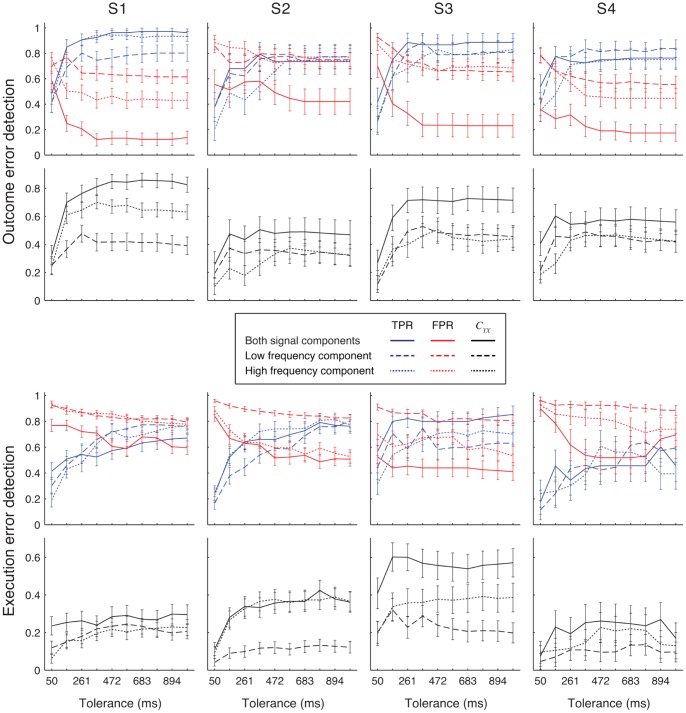
Overview of detection results. Detection accuracy was measured when signals from all ECoG electrodes were used for detecting outcome (top half) and execution (bottom half) errors. For detection, we used the low frequency component (dashed line), the high frequency component (dotted line) or both frequency components together (full line). Top rows of panels show the *TPR* (blue lines) and *FPR* (red lines), while the bottom rows of panels show the normalized mutual information *C_YX_* (black lines). Error bars show 95% confidence intervals. Different columns show results for different subjects. Detection was made using rLDA.

**Figure 8:**
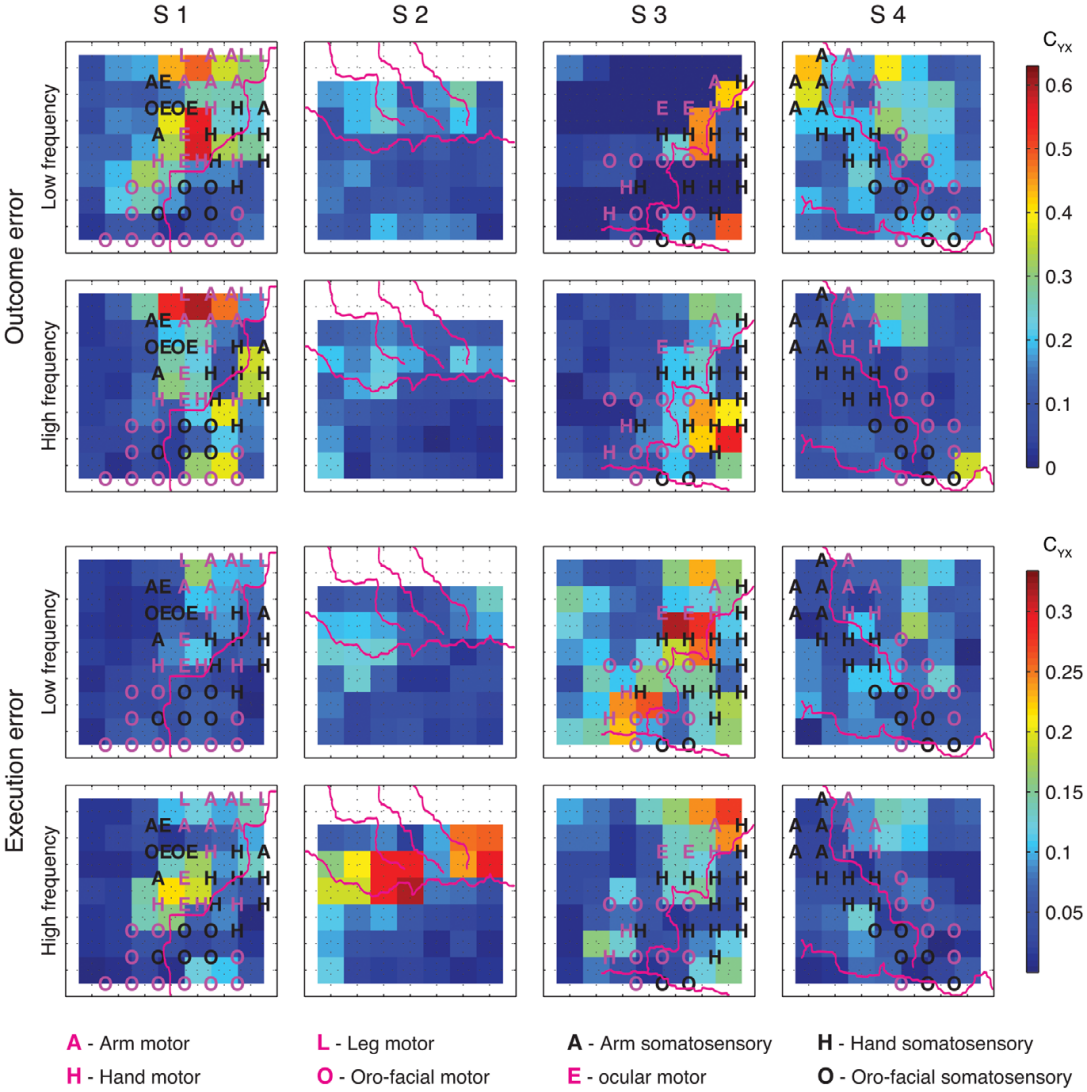
Spatial distribution of *C_YX_* for detection of outcome and execution errors. Detection was performed by using either low or high frequency components of the signals recorded from electrode quartets (2×2 neighboring electrodes). Purple lines depict the central sulcus, the Sylvian fissure and, for S2 only, the pre and post central sulci. Letters in the squares mark the functional subarea (A – arm, H – hand, L – leg, E – ocular, O – oro-facial) in motor (purple) and somatosensory (black) cortex as determined by ESM. Every coloured square represents one quartet of electrodes with the electrodes at the corners of the square. Colours of the squares depict the normalized mutual information according to the colour bar. Since no recordings were made from the top row of grid electrodes for S2, we show the top row of quartets as white. The top left square in the ECoG grids correspond to the electrode closest to the red star in Figure 3. Detection was made using rLDA.

To quantify how well outcome and execution events can be detected, we used signals from all ECoG grid electrodes and in both signal components as an input for our detection algorithm (Figure 6, 7, see Figure 8 for topographical distribution of signals informative for error detection). When detecting outcome error with a tolerance of 366 ms and across all four subjects, average *C_YX_* was 0.69±0.04 with average *TPR* of 0.87±0.03 and average *FPR* of 0.24±0.04 (for individual subject values see Table 3). For detection of execution errors with the same tolerance, average *C_YX_* was 0.33±0.03 with average *TPR* of 0.64±0.04 and average *FPR* of 0.61±0.03 (for individual subject values see Table 3). Over all tolerance values, outcome error *C_YX_* values were higher than execution error *C_YX_* when both frequency components were used (*C_YX_* difference for tolerance of 366 ms: 0.36±0.05; for individual subjects: S1∶0.58±0.08, S2∶0.17±0.11, S3∶0.15±0.12, S4∶0.30±0.13).

**Table 3:** Detection accuracy.

	Outcome error				Execution error			
	S1	S2	S3	S4	S1	S2	S3	S4
*C_YX_*	0.82±0.06	0.50±0.09	0.72±0.09	0.55±0.09	0.24±0.05	0.33±0.05	0.57±0.08	0.25±0.10
*TPR*	0.92±0.04	0.79±0.09	0.87±0.08	0.73±0.08	0.52±0.07	0.66±0.06	0.81±0.07	0.43±0.12
*FPR*	0.13±0.05	0.58±0.08	0.23±0.09	0.24±0.08	0.73±0.05	0.62±0.05	0.46±0.07	0.56±0.12

Detection accuracy when both LFC and HCF signals from all ECoG electrodes are used for detection using rLDA.

Over all subjects, using both signal components gave significantly higher *C_YX_* then when using either one of the components alone for all tolerance values (*C_YX_* difference for tolerance of 366 ms over all subjects: outcome error: LFC & HFC vs. LFC: 0.23±0.05; LFC & HFC vs. HFC: 0.16±0.05; execution error: LFC & HFC vs. LFC: 0.15±0.04; LFC & HFC vs. HFC: 0.05±0.04). Using HCF gave significantly higher *C_YX_* than using LFC for tolerances of 366 ms and higher (*C_YX_* difference for tolerance of 366 ms: outcome error: 0.07±0.05; execution error: 0.09±0.03).

### Topographical Distribution of Informative Signals for Error Detection

To determine the topographical distribution of signals that were informative for the error detection, we performed detection using signals recorded from electrode quartets (Figure 5). For most of the subjects, several isolated, often spatially quite distant peaks of *C_YX_* could be found over the cortical regions we recorded from. Locations of these peaks often differed for different error types and different signal components. Thus, we did not notice any topographical location that was systematically beneficial for detecting either outcome or execution errors.

### Error Detection Using Signals from Motor or Somatosensory Areas

**Figure 9:**
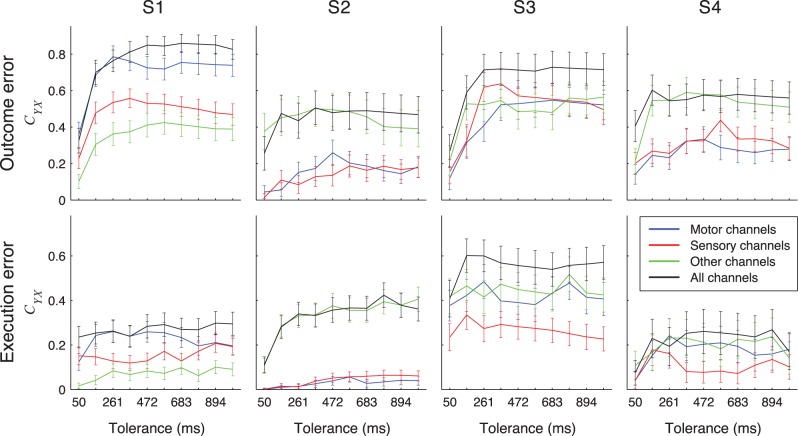
Detection results when using signals from anatomical electrode subsets. *C_YX_* for detecting outcome (top) or execution (bottom) errors using signals recorded from electrode subsets containing all electrodes over motor cortical areas (blue lines), somatosensory cortical areas (red lines), all other remaining electrodes (green lines) and all electrodes together (black lines). Error bars show 95% confidence intervals. Detection was made using rLDA classifier.

We compared the performance of error detection based on recordings from different anatomical subareas (motor, somatosensory and other areas) to the error detection performance based on recordings from all channels (Figure 9). For all 4 participants, the detection performance was highest when all electrodes were used; with subarea electrode sets reaching in some cases an equivalent performance.

Next, we tested if recordings from motor or somatosensory areas provided enough information for high accuracy of error detection. To this end, we calculated the percentage of *C_YX_* achieved when using signals from these areas compared to the *C_YX_* values achieved when using all ECoG grid electrodes. These percentages were first averaged over all tolerance values and then over subjects. For S2, motor and somatosensory cortex was not well covered with electrodes as only 3 or 2 electrodes recorded from these areas (Table 4). We, therefore, excluded S2 from this analysis. Detection performance from motor cortex signals was 75±2% and 77±4% of the total detection performance for outcome and execution error respectively. Performance from somatosensory cortex signals reached 63±2% (outcome error) and 50±3% (execution error) of the total performance.

**Table 4:** Number of electrodes belonging to different anatomical subsets.

	S1	S2	S3	S4
Motor	22	3	18	11
Somatosensory	18	2	11	14
Other	20	51	35	39

Number of electrodes belonging to motor, somatosensory and other anatomical subsets for each of the subjects.

### Detection from Smaller Electrode Sets

**Figure 10:**
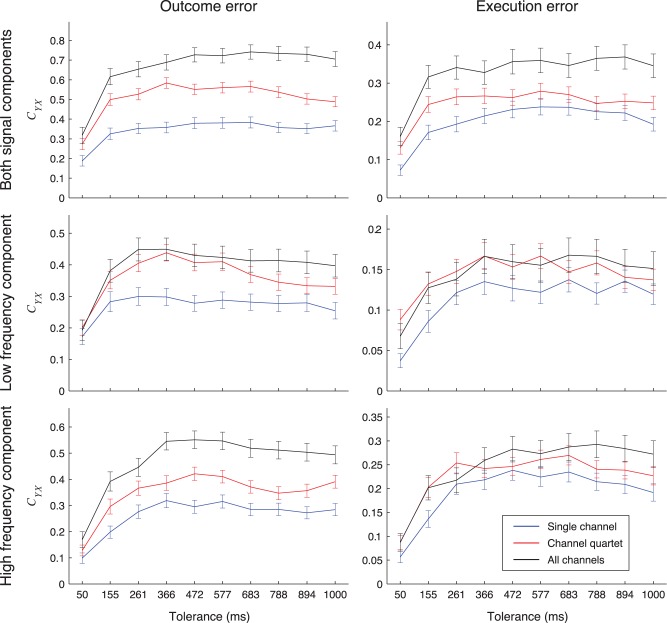
Detection results when using signals from electrode sets of different sizes. *C_YX_* for detection of outcome (left) or execution (right) errors for different sizes of the electrode sets, maximized over all possible electrode subsets and averaged over subjects. We compared single electrodes (blue lines), electrode quartets (red lines) and the set of all grid electrodes (black lines) when using low frequency component (bottom), high frequency component (middle) or both frequency components (top) of the recorded signals. Detection was made using rLDA. Error bars show 95% confidence intervals.

We investigated whether one can detect error events with smaller electrode subsets with accuracy similar to detection when all ECoG grid electrodes were used (Figure 10). When both frequency components were used for the tolerance of 366 ms, maximum *C_YX_* from single electrodes was 60±6% for outcome error and 66±9% for execution error of the *C_YX_* when all electrodes were used. For electrode quartets and both frequency components, maximum *C_YX_* was 87±6% for outcome error and 78±10% for execution error of *C_YX_* when all electrodes were used.

### Effect of MRNR Subtraction on the Normalized Mutual Information

**Figure 11:**
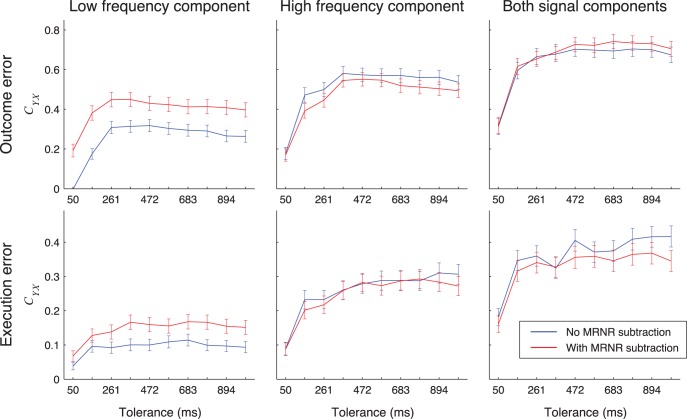
Detection results when using signals with or without MRNR subtraction. *C_YX_* for detection of outcome (top) or execution (bottom) errors when using signals with (red) and without (blue) MRNR subtraction from all electrodes. Different columns show *C_YX_* when low frequency component (left), high frequency component (center) or both signal components (right) of the recorded signals were used for detection. Error bars show 95% confidence intervals. Detection was made using rLDA.

We also investigated whether MRNR subtraction affected the detection of the error events (Figure 11). Over all subjects and tolerance values, the difference in *C_YX_* when MRNR subtraction was and was not used was not significant when both LFC and HFC were used for detection, except for tolerances of 472 ms and 1 s for execution error for which the signals without MRNR subtraction gave higher *C_YX_* values (*C_YX_* difference for tolerance of 366 ms: outcome: −0.01±0.05; execution: 0.00±0.04). When only LFC was used for detection, using MRNR subtraction lead to a slight, but significant improvement, except for execution error for tolerances of 155 ms and 261 ms (*C_YX_* difference for tolerance of 366 ms: outcome error: −0.14±0.04; execution error: −0.07±0.03). For the high frequency component, using MRNR subtraction did not change the *C_YX_* values, except for outcome error for tolerances of 155 ms and 894 ms, for which the *C_YX_* values were slightly significantly worse (*C_YX_* difference for tolerance of 366 ms: outcome error: 0.03±0.05; execution error: 0.00±0.04).

### Selection of the Classifier Type for Detection: rLDA vs. rQDA

**Figure 12:**
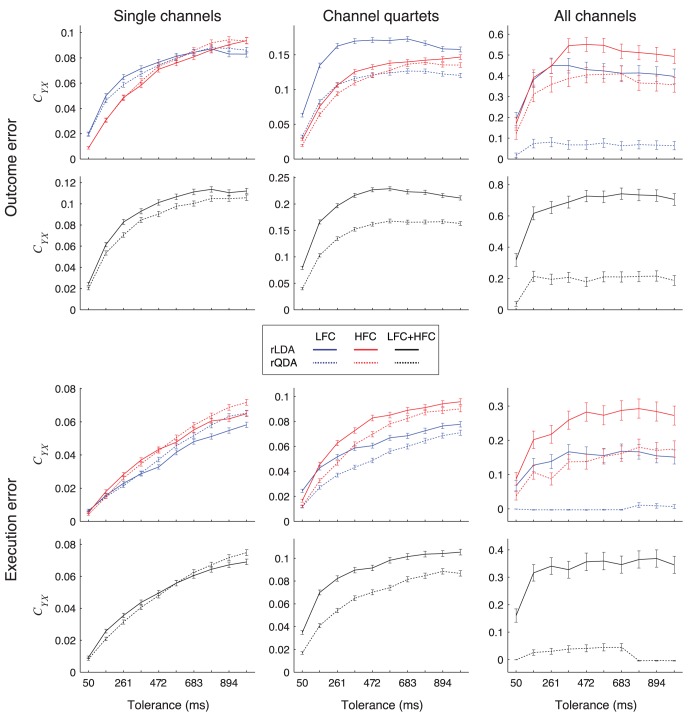
Detection results when using rLDA or rQDA classifiers. *C_YX_* for detection of outcome (top) or execution (bottom) errors for different electrode set sizes, using LFC (blue lines), HFC (red lines) or both components together (black lines) and for rLDA (full lines) and rQDA (dotted lines) based detection algorithms, averaged over all subjects and all possible electrode subsets. Error bars show 95% confidence intervals.

We compared the detection performance between rLDA and rQDA (Figure 12). rQDA is more flexible, but has the drawback that more free parameters need to be estimated from the training dataset. The number of free parameters is a quadratic function of the number of signal features, which, in turn, is the product of the number of electrodes and the number of features taken from each single electrode. Therefore, if the dataset is quite large and the total number of features used to build the classifier is quite small, rQDA might outperform rLDA. On the other hand, if the data is limited and the total number of features is high, rLDA might outperform rQDA. We wanted to determine in which one of these two regimes our dataset was.

If signals from single electrodes were used for detection, rLDA and rQDA yielded essentially the same performance, with differences being small compared to the performance of any of the classifiers and, in many cases, insignificant (Figure 12). If signals from electrode quartets were used, rLDA performance was in most cases significantly higher than rQDA, but always at least as high as rQDA. For all electrodes, rLDA always significantly outperformed rQDA.

## Discussion

In our previous study [16] we demonstrated that the ECoG signals used here for error detection indeed reflect outcome and execution ERNRs and are not related to movements or caused by visual stimuli or surprise effects. Here, we showed that both outcome and execution error events can be reliably detected from continuous neuronal activity measured with ECoG electrode implants. These results suggest that, for both error types, it is possible to detect more than half of the errors and that the number of false positive detections is comparable to the number of true positive detections. In our experiment, error events were extremely rare. Therefore, these results suggest that error events can be detected with high reliability. This notion is supported by the relatively high values of the normalized mutual information.

Signals informative about the times of error events were not found in one isolated cortical area only, but rather in multiple different areas (Figure 8). Often, it was possible to find several, mutually isolated *C_YX_* peaks in one subject for the same error type and the same frequency component. In addition, *C_YX_* peaks often had different locations for different error types and different location for different frequency components. This suggests that neuronal responses coding for outcome errors might be independent from the neuronal responses coding for execution errors. In addition, it suggests that low and high frequency component of the neuronal responses coding for the same error might also be independent. This is consistent with results in the previous study by Milekovic et al. [16], where it was shown that the topographical distribution of ERNR’s signal to noise ratio had similar, distributed properties.

We also tested whether signals from a particular anatomical area gave more or less informative signals for detection. To this end, we divided the ECoG electrodes for each of the subjects into three subsets: motor set, somatosensory set and the ‘other’ set. In S2, the ECoG grid was implanted more ventrally than in other subjects, thereby covering only the ventral motor and somatosensory areas. In addition, ESM was not performed on S2. Therefore, for S2, we had to use anatomical reconstruction of major cortical sulci locations from the patients’ MRI scans to determine which electrodes belonged to motor and somatosensory subsets. This resulted in a motor set of only 3 electrodes and a somatosensory set of only 2 electrodes (Table 4). Due to the low number of electrodes in these subsets, and due to the more ventral location of the ECoG implant, we decided that detection results of motor and somatosensory sets for S2 were not representative as these areas were not sufficiently covered and, therefore, excluded S2 from the anatomical area specific analysis. For the remaining three subjects, none of the anatomical subsets provide better signals for detecting outcome errors. In the case of execution error, detection using motor and other electrode subsets was similar and only a bit better then detection from the somatosensory subset.

A complete 8×8 electrode ECoG grid covers a surface of around 64 cm^2^. To implant such grids, a craniotomy of similar size is required. Studies on risk factors of subchronic implantations for pre-neurosurgical epilepsy diagnostics indicate that the size of the implant is a risk factor for complications of subdural ECoG implantations [39]. Therefore, we investigated whether similar detection accuracy could be obtained using signals from smaller parts of the grid only. Signals from single electrodes gave much lower detection accuracy in comparison to using all ECoG electrodes but, nevertheless, more than 60% of the *C_YX_* value when using all grid electrodes could be obtained for any of the error types. Detection based on signals from electrode quartets (2×2 neighbouring electrodes) almost reached the level of detection obtained by using signals from all electrodes, obtaining more than 78% of the *C_YX_* value when using all grid electrodes for any of the error types. These results suggest that, if the optimal location for an implant is known in advance, one could safely reduce the size of the electrode by 16 fold, with only a small loss in detection accuracy.

Besides reducing medical risks, there are additional benefits when reducing the number of electrodes used for error detection. The numerical complexity of the detection algorithm is a quadratic function of the number of used electrodes. Therefore, reducing the number of electrodes by 16 fold could reduce the numerical complexity of the detection algorithm by up to 256 times, which would drastically reduce the computational requirements of the detection process, allowing the process to be run on less powerful computers, or freeing computational resources for other tasks. To determine which electrodes or electrode quartets would be best for detection, one could run the entire detection process on a set of data recorded for such calibration purposes. On the other hand, such an optimization process might take a considerable amount of computation time.

A high proportion of our electrodes were located above the motor and somatosensory cortical areas. Therefore, it should be expected that some fraction of our signals contained movement related neuronal signals. In Milekovic et al. [16], analysis revealed that large amounts of the variance on some of the electrodes could be explained by a classifier predicting neuronal signals from eye and hand movements. This study than removed this proportion of the signals by subtracting the movement related signals predicted by such classifiers. Here, we performed error detection using the same MRNR subtraction procedure and compared it to the detection accuracy when error detection was performed without MRNR subtraction. We found that, on average, MRNR subtraction did not significantly change the detection accuracy, demonstrating that our error detection was really based on error related and not on any movement related neuronal signals.

We also compared detection accuracy between detection using rLDA and rQDA. In our experiment, subjects performed during about one hour of recordings, which left about 20 minutes of recordings for training the detector. In these experimental conditions, the detector using rLDA clearly outperformed the detector using rQDA when signals from all ECoG electrodes were used. In this case, the number of possible features used for detection greatly outnumbered the number of ERNR examples used for building the classifier. Since rQDA classifiers need to estimate a higher number of parameters than rLDA classifiers, it is no surprise that detection based on rLDA showed better results. On the other hand, when signals from only a single electrodes and a single signal component were used for detection, using rQDA was as accurate or better than using rLDA. In this case of low numbers of features, the detection benefited from the higher flexibility of rQDA classifiers. But, when using signals from only 4 electrodes, detection accuracy was significantly higher when using rLDA. For larger datasets containing more ERNR examples that could be used for detection classifier building, rQDA might further outperform rLDA classifiers although, based on our studies, one would rather include larger number of channels since, by doing so, the gain of detection accuracy becomes higher.

### Characteristics of Signal Components Used for Detection

Here, we used both low (0–8 Hz) and high (60–128 Hz for S1 and S2 and 60–200 Hz for S3 and S4) frequency components of the neuronal signals to detect the error events. According to the study of Milekovic et al. [16], these signal components gave two different, possibly independent, sources of information about errors. Other studies showed that neuronal responses with similar spectro-temporal characteristics can be evoked by non-error events, such as different movements [40]–[46], somatosensory and auditory stimuli [47]–[49], word recognition [50], face recognition [51] and attention and short term memory [52]. One could argue that detecting ERNR in a more natural environment compared to our, highly controlled task will be more challenging as the ERNRs might not be differentiable from neural responses to non-error events. This should, however, not necessarily hinder the applicability of our error detection appoach. If subjects are focused on the task at hand, they are expected to perform only minimal amounts of additional movements, receive minimal amounts of additional tactile stimuli and will probably not perform additional cognitive tasks. In our task, subjects were merely asked not to move too much and to try to remain focused on the task. We argue that they would show similar behavior if they were motivated to perform the task for their personal benefit, such as navigating the cursor or artificial hand towards the target. Therefore, we think that the ECoG signal can also be applied for online continuous error detection under more natural conditions.

Detection of error events could still be possible, even in environments with more somatosensory stimuli and more different tasks at once. ERNRs used in this study were evoked on multiple, often quite distant, electrodes and these evoked responses exhibited quite different time courses. This makes the ERNR responses highly redundant and likely differentiable to other, non-error neuronal responses. Some of the earlier mentioned studies already used these signal properties to differentiate between neuronal responses to different non-error events [40], [41], [49] and the same principle could work for ERNRs as well. Further studies are required to test the accuracy of error detection in such more complex environments.

### Comparison to Previous Detection Studies

Several studies investigated the detection of epileptic seizures from neuronal recordings [53], [54]. Since epileptic seizures occur very rarely and cause hospital staff alarms during the epileptic assessment periods, there is a strong requirement to keep the number of false positive detections at a minimum. Therefore, when measuring the accuracy of seizure detections, *TPR* is usually combined with the number of false positives per hour [55]. Since the frequency of decoding errors during continuous control with current BMI approaches is much higher than the typical number of epileptic seizures, using the number of false positives per hour as a measure of performance does not apply well to error detection. A number of other studies used neuronal signals to detect movement related events, such as movement onset [56]–[59], movement planning phase [60] and periods of movement related synchronization and desynchronization [27]. These studies mainly used *TPR* and *FPR* to visualize the detection results, while some of them used the Youden index *i* = *TPR*-*FPR* [61] as a single measure of detection performance [56], [59]. Solis-Escalante et al. [27] used mutual information to report their final results, but still used the Youden index to calibrate their detector. Here, due to its strong theoretical foundations, we used normalized mutual information to both calibrate the detector and measure its accuracy.

The afore mentioned studies used a wide variety of algorithms for detection: expectation maximization Gaussian mixture classifier [59], k nearest neighbors [57], linear discriminant analysis [57], local field potential β-band power threshold crossing [58], cross-correlation threshold crossing [56], support vector machines [27], recursive Bayesian classifier [60] and phase slope index threshold crossing [54]. Here, we used regularized versions of linear and quadratic discriminant analyses with a variable threshold for detection. This makes our algorithms linear or quadratic in nature. Use of more complex and flexible algorithms might improve the detection, although, in the present study, the simpler rLDA algorithm outperformed the more complex rQDA algorithm whenever more than one electrode or signal component was used.

The afore mentioned studies detecting movement states did not investigate the effect of temporal tolerance on the precision of the detector. Here, we showed that detection accuracy rises with the tolerance, until it saturates at around 300–500 ms. This implies that ERNRs used to detect error events are not perfectly timed to those events, and that the variability or ERNRs in response to error events is at the level of 300–500 ms. Reasons for this variability might be imprecision due to noise or other kinds of neuronal activity (e.g. ongoing activity) or the variability in the time subjects needed to recognize the error. Measurements of choice reaction time variability [62] have reported reaction time variability of about 400 ms (95% of all reactions). In any case, the variability caused by the limited detection frequency of 32 Hz should be negligible compared to other effects.

### Comparison to Previous ERNR Studies

Most of the earlier ERNR studies concentrated on activity of the anterior cingulate cortex (ACC) and its functional significance [63]–[65]. These studies were conducted using trial-based tasks measuring brain activity by the electroencephalogram (EEG). The observed neuronal responses were classified into several types: response error related negativity (rERN) [66], [67], feedback error related negativity (fERN) [68], observation error potential (oErrP) [69] and interaction error potential (iErrP) [70]. In contrast to these studies, we investigated errors during a continuous task. At this stage, it is unclear what the relation between the mechanisms generating these two kinds of responses is or whether the mechanisms are different at all. Krigolson and Holroyd [15], [18] compared EEG correlates of error-related activity during a continuous tracking task to the ERNs observed in trial-based tasks of previous studies [67], [68]. Krigolson and Holroyd report similarities (e.g. spatial distribution of the neuronal error signal) and differences (e.g. in the timing of the response) between the continuous error responses recorded in their study and trial-based rERN and fERN reported in other studies. However, in contrast to our study they use EEG instead of ECoG and their continuous task is also different from ours. Thus, further investigations would be needed to clarify the relation between the ERNRs reported in our study and the well-established ERN. To address this interesting question, one could compare trial-based and continuous error responses in the same subjects using the same recording methodology. To the best of our knowledge, no such study has been carried out so far and we consider this to be an interesting topic for future research.

ERNRs have also previously been found in motor cortex [14], [16], [69], [71], somatosensory cortex [14], [16] and in other cortical areas [14], [16], [72]–[77]. Our study extends our previous findings [16] by showing that error related neuronal responses recorded by ECoG can be detected in continuous neuronal activity recordings. Furthermore, we demonstrated that outcome errors could be detected with higher accuracy than execution errors.

### Relevance for Brain Machine Interfaces

One motivation for this study was to investigate whether ERNRs can be used to improve the performance of continuous movement BMIs [1], [3], [8], [9], [78]–[80].

To achieve this goal, (i) error related neuronal signals need to be present in the used recording of neuronal activity, (ii) these error related signals need to be detectable, (iii) different error types (e.g. execution and outcome error) need to be differentiable from the signals and (iv) adaptive decoding algorithms utilizing error signals need to be available. Extensive research in error related neuronal signals already showed that such signals can be recorded with a wide range of recording techniques. Specifically, Milekovic et al. [16] showed that error signals can be recorded using ECoG during continuous tasks and that execution errors can be differentiated from outcome errors, thereby resolving points (i) and (iii) for ECoG based BMIs. Adaptive algorithms decoding continuous movements can indeed benefit from error detection [12], [81], resolving point (iv). In this study, we showed that error detection is possible, thereby resolving point (ii). Even though all points have now been resolved, it still remains necessary to demonstrate the proposed continuous BMI decoding system that utilizes neuronal error signals in an online study, making this an interesting topic for future research.

Our previous study [16] showed that outcome and execution errors can be reliably distinguished upon detection. Instead of just providing binary information, whether a decoding error has been made or not, detection of multiple error types could further improve BMI operation. Outcome error detection can be used to subsequently correct the errors [5]–[7], [70], [82]. Subsequently correcting decoded trajectory errors by detecting execution errors might not be beneficial, since BMI users will rather try to correct the decoding mistake by corrective movements. On the other hand, in a task where movements are made to perform some kind of selection, e.g. by guiding a cursor to one of several target locations, it would be possible to subsequently correct incorrect selections by detecting outcome errors, thereby making the BMI more efficient. In addition, outcome errors can also be used within the reinforcement learning framework [83]. However, reinforcement learning algorithms tend to require long recording sessions, making them much slower in their adaptation towards more efficient BMI decoders.

Detection of execution errors can be used to directly label incorrectly decoded trajectories and, thus, the part of the recordings that were incorrectly interpreted. Such information can be used to facilitate BMI algorithm adaptation [12], [81].

Even though we carried out offline analyses in this study, our detection methods are directly applicable to detect errors in online experiments and could be utilized as a part of an online BMI system. For intermediate channel and feature numbers no special computer hardware will be required; in fact similar preprocessing and decoding algorithms [23], [84]–[86] as utilized by our detection algorithm, have already been used in real-time during online BMI applications on standard computing hardware. For high channel/feature numbers parallel processing, e.g. utilizing the graphics processing unit or multiple cores of standard laptop/desktop computers, might be used [87].

For most invasive BMIs, motor cortex is the primary target area for the implantation of electrodes whose signals are to be used to extract intended motor actions. We demonstrated that one can detect error signals with high accuracy based on motor cortical signals only. Therefore, movement decoding and error detection may be implemented using the same electrode implants. Consequently, no additional implants over other cortical areas would be required for BMIs employing such neuronal error signals, thereby not inflicting additional medical risk when adding error detection to a BMI system.
